# Diet and Lifestyle in the Spanish Population and Their Relationship with Sociodemographic Variables: A Descriptive Study

**DOI:** 10.3390/foods12183409

**Published:** 2023-09-13

**Authors:** Elena Sandri, Eva Cantín Larumbe, Roser Part-Ferrer, Javier Ferrer-Torregrosa, Nadia Fernández-Ehrling

**Affiliations:** 1 Doctorate School, Catholic University of Valencia San Vicente Mártir, 46001 Valencia, Spain; elena.sandri@ucv.es; 2 Faculty of Medicine and Health Sciences, Catholic University of Valencia San Vicente Mártir, c/Quevedo 2, 46001 Valencia, Spain; 3 Escuela Técnica Superior de Ingeniería Informática, Polytechnical University of Valencia, Camí de Vera s/n, 46022 Valencia, Spain; ecanlar@etsinf.upv.es; 4 Podiatry Department, Faculty of Medicine and Health Sciences, Catholic University of Valencia San Vicente Mártir, C/Ramiro de Maeztu, 14, 46900 Torrent, Spain; roser.part@ucv.es (R.P.-F.); nadia.fernandez@ucv.es (N.F.-E.)

**Keywords:** nutrition, Spain population, socioeconomic factors, physical activity, lifestyle

## Abstract

A healthy lifestyle and proper nutrition have a major impact on the well-being of a population. Therefore, the aim of this research is to describe the behavior of these habits in relation to sociodemographic variables to provide data on the development of effective training and awareness-raising actions. Methods: An observational, descriptive and cross-sectional study was carried out. To study the behavior of a series of variables related to eating habits and lifestyle, a questionnaire was designed and validated and subsequently disseminated online, by means of nonprobabilistic snowball sampling, relying on social networks. The sample collected consisted of 18,070 young adults of Spanish nationality. Bivariate comparative analyses were performed using *t*-test independent samples, and the effect size (ES) was calculated by determining Cohen’s D coefficient. A multivariate analysis were conducted using linear regression and principal component analysis. Results: Adults eat better but have a poorer quality of rest and are more sedentary than young people. No gender differences were found in nutritional habits; however, men engage in more sports and sleep better. People with a higher educational level have better nutritional and sleep habits, but are more sedentary, as are people of a higher socioeconomic level. Conclusions: Higher socioeconomic and educational levels seem to favor a healthier lifestyle. The Spanish population aged 18–45 years needs to make dietary changes but leads an active lifestyle.

## 1. Introduction

Lifestyle is a determinant of good health and well-being, which includes diet, physical activity, rest and other personal habits [1,2,3]. The lifestyle of a population depends on sociodemographic factors such as age, sex, education, occupation and income [4,5,6,7]. The study of lifestyle provides necessary information to guide health-promoting initiatives tailored to the needs of a population. Various instruments exist assessing singular facets of lifestyle, such as diet [8,9,10], physical activity [11,12,13] or alcohol use [14,15]. However, comprehensive tools evaluating multiple lifestyle domains are lacking. We developed the NutSo-HH questionnaire to assess lifestyle habits and sociodemographic factors in the Spanish population.

This study focused on young adult Spanish population since youth and early adulthood are sensitive and important periods during which significant changes occur in individuals, such as rapid weight gain [16] or the development of unhealthy nutritional behaviors and habits throughout life [17,18]. Therefore, it is important to have a thorough knowledge of the habits of this population to plan public health actions.

This study aims to elucidate associations between sociodemographic factors and lifestyle. Specifically, we examined age and gender, education level and purchasing power in relation to diet quality, physical activity, sleep, smoking, alcohol use and nightlife habits. Understanding connections between sociodemographic variables and lifestyle provides evidence to inform public health policies and campaigns. Tailored initiatives resonate better with target populations compared to one-size-fits-all approaches [19,20].

The literature shows that educational level has a significant impact on healthy lifestyle choices [21]. In general, people with higher education are less likely to abuse alcohol, exercise more and eat healthier foods than the average population [22]. Education and health literacy strongly influence healthy behaviors, and people with lower levels of education have shorter lifespans or spend more years of life with physical limitations or metabolic diseases [23]. In addition, identifying the links between income and nutrition guides the allocation of resources, such as subsidizing healthy foods in low-income areas [18,19]. Recognizing the links between education and physical activity informs advocacy strategies to increase exercise among less educated groups [20,21].

Likewise, understanding the patterns of lifestyle habits in terms of age and gender, allows health promotions and more personalized education and awareness campaigns to be carried out for a specific population. Revealing connections between lifestyle and sociodemographic factors enables the development of appropriately targeted, culturally-relevant, evidence-based health promotion activities. While previous research has studied some of these associations in isolation in Spain, our study is the first to simultaneously analyze this complex network of predictors and their effects on diet and physical activity patterns at the population level. By revealing unique sociodemographic profiles associated with healthy and unhealthy behaviors, our findings have important implications for the development of public interventions and health policies focused on groups with the greatest needs. Furthermore, the large sample used and methodological rigor in data collection and analysis ensure the validity of our results and their ability to provide a solid foundation for future studies in this area.

There are three main objectives in this study: (a) to examine if there is a relationship between age and sex and lifestyle habits, (b) to analyze the influence of education and purchasing power of the Spanish population on lifestyle habits, and finally, (c) to study the relationships between lifestyle habits and to observe which ones differentiate the Spanish young adult population.

## 2. Materials and Methods

### 2.1. Subjects

A sample of n = 18,070 young adults of Spanish nationality (age: 18–45 years) was collected, and a prospective cross-sectional study was carried out; respondents who did not provide food for themselves, such as those hospitalized or incarcerated, were excluded.

### 2.2. Instruments and Variables 

To collect data for the present study the research team developed and validated the NutSo-HH scale, an instrument able to measure different aspects of the lifestyle of the Spanish population.

The Nutritional and Social Healthy Habits Scale (NutSo-HH) was developed and validated in two phases: the first phase followed European recommendations [24] in terms of conceptualization, design, cognitive and expert review and pilot testing; the second phase was applied to a sample of 571 young Spanish adults through nonprobabilistic sampling in social networks. The NutSo-HH consists of 23 items assessing eating habits, physical activity, sleep and alcohol consumption. Confirmatory factor analysis, validity analysis, comparison with other instruments and reliability analysis were performed, and the scale showed adequate psychometric properties. The omega coefficients [25] ranged from 0.521 to 0.815.

We used four sociodemographic variables in this study: sex, age, level of education and income level. Regarding the sex variable, a distinction was made between women and men; concerning the age variable, a distinction was made between young people aged 18 to 30 years old and adults aged 31 to 45 years old; with respect to the educational level variable, subjects with basic studies were considered to be those who had studied up to the level of Baccalaureate or Vocational Training, while subjects with at least a degree were considered to be those with higher studies. Finally, regarding the income level variable, low income was defined as the income of the family nucleus below EUR2200 gross/monthly, and high average income was that above this amount [26].

A reduced version of the IASE index (Índice de Alimentación Saludable para la población española) [27] was used to study nutritional health. This index is based on the frequency of consumption of certain food groups as well as on the variability of the foods eaten. The index used has a maximum score of 73, obtained from the sum of the scores of each variable. To score each variable, a maximum score of 10 was assigned to those dietary habits in accordance with the recommendations proposed by the Spanish Society of Community Nutrition (SENC) [28] and then scaled down to a minimum score of zero for those habits that significantly differ from the SENC recommendations.

For lifestyle variables and for nutrition variables not included in the IASE, such as Sedentary lifestyle, Fast-food consumption, Consumption of fried foods, Consumption of ultra-processed food, Sleeping hours, Getting up rested, Sleep quality, Smoking, Alcohol consumption, Getting Drunk and Night outings were categorized on a Likert scale from 1 to 4 points, following the criteria indicated in Table 1, except for Physical activity, as it was used as a numerical measure.

### 2.3. Data Collection

The questionnaire was distributed telematically between August 2020 and November 2021, using the support of the Google Forms program, using nonprobabilistic snowball sampling. It was distributed through social networks (@elretonutricional), WhatsApp and email to different establishments throughout Spain. 

### 2.4. Statistical Analysis

Fisher’s exact test was applied to determine if the two categories of each sociodemographic variable were associated. It was established a threshold of 0.01 for the *p*-value. Independent samples were *t*-tested to compare the categories of sex, age, educational level and income level.

For each habit variable, the Cohen’s d statistic is shown, which is a measure of effect size that indicates the magnitude of the difference between the groups. A positive value indicates a significant difference, with the first group having a higher mean than the second group, while a negative value indicates the opposite. In addition, the 95% confidence interval for the mean difference between the groups is provided. This interval shows the range within which the true mean difference is expected to lie with a 95% confidence level.

The magnitude of differences was assessed using the Cohen’s d effect size, which was interpreted using the following rating scale: trivial (0.00–0.09), small (0.10–0.59), moderate (0.60–1.19), large (1.20–2.00) and very large (>2.00) [29]. The level of statistical significance was stablished at *p* < 0.05. All analyses were performed using statistical analysis software (JASP, Holanda, Países Bajos) and RStudio 4.3.0. 

## 3. Results

**Figure 1:**
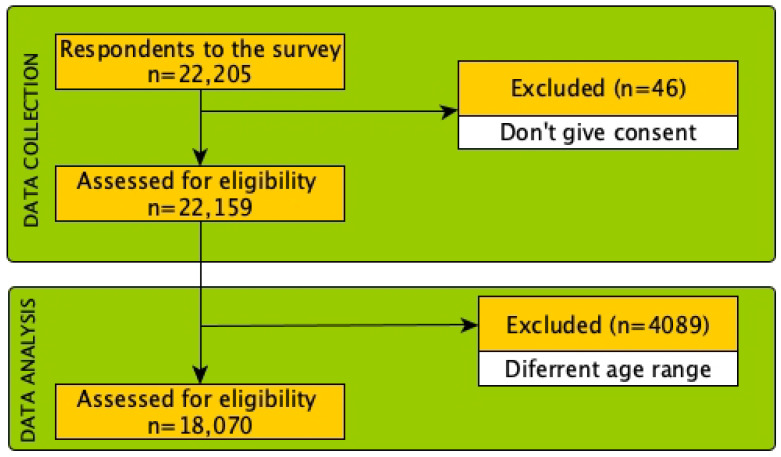
Flow diagram of the selection and analysis process of the respondents included in this study.

The final analytical sample consisted of 18,070 respondents, including 3200 men and 14,870 women, with a mean BMI of 23.73 ± 4.67. Sociodemographic characteristics can be seen in Table 2. However, 4135 respondents did not meet the proposed age range or did not give their consent to participate in this study (see Figure 1).

Table 3 shows the mean and deviation, providing information on various aspects related to the lifestyle habits of the different demographic groups. We highlight the Healthy Eating Index (IASE) which shows an increase in the mean score of adults with higher education (Educational level), 54.46 ± 9.48, compared to those with basic studies, 52.00 ± 10.38.

Similarly, we observed differences in the mean number of minutes dedicated to physical activity. Males and adults (31–45 years) had higher levels of physical activity compared to females and young people (18–30 years).

As for the consumption of fast food, fried food and ultra-processed food, no differences were found between the groups.

We also observed that young people (18–30 years) slept slightly more than adults (31–45 years), although there were no significant differences between demographic groups. Sleep quality was rated slightly higher in males (3.51 ± 0.96) than in females (3.39 ± 1.02).

Males had a higher frequency of alcohol consumption (1.82 ± 0.88) compared to females (1.67 ± 0.80), with no differences in drunkenness between the two. It was shown that young people (18–30 years) had a slightly higher frequency of night outings (1.30 ± 0.51) compared to adults (31–45 years) (1.09 ± 0.31), and that it was also higher in men (1.24 ± 0.48) compared to women (1.20 ± 0.44).

Analyzing the nutritional habits adopted in relation to income level, we found that people with a middle- or high-income level had a significantly higher IASE score compared to people with low income (54.70 ± 9.49 vs. 52.75 ± 10.13). 

Table 4 presents the results for each variable related to habits, along with the corresponding values of the Cohen’s d coefficient and confidence intervals. Confidence intervals indicate the likely range within which the true mean difference lies. 

The sex variable exhibits a moderate effect size compared to physical activity (d = −0.74). Conversely, excessive alcohol consumption (d = −0.22) demonstrates a small effect size.

Regarding age, small effect sizes are observed for fast food consumption (d = 0.22), consumption of fried foods (d = 0.26), hours of sleep (d = 0.20), sleep quality and night outings (d = 0.48).

When analyzing the Healthy Eating Index (IASE), small effect sizes are evident for educational level and income level (d = −0.25 and d = −0.20, respectively), as well as for educational level and the smoking variable (d = 0.20).

**Figure 2:**
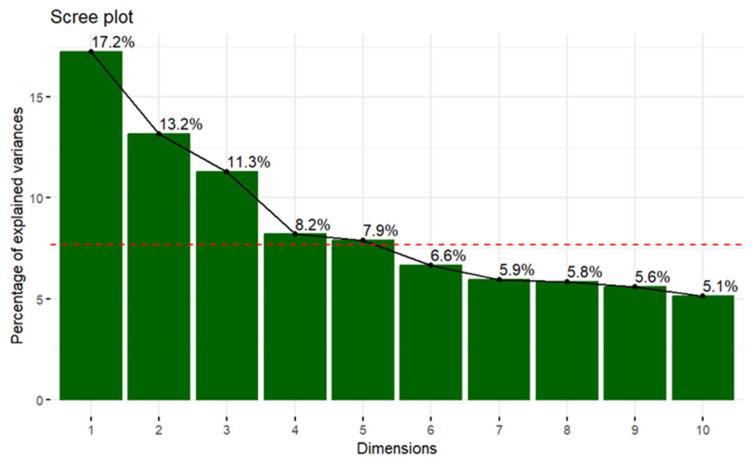
Distribution of the contribution of each dimension to the model (NOTE: The red line represents the average percentage of variance explained by the model).

Subsequently, it was proposed to study the relationships between ordinal numerical variables and to see which ones differentiate the Spanish young adult population using the principal component analysis (PCA) method to reduce dimensionality and not to perform analyses for each pair of variables. In the analysis, two principal components were selected, explaining 30.4% of the variability of the data, as shown in Figure 2.

**Figure 3:**
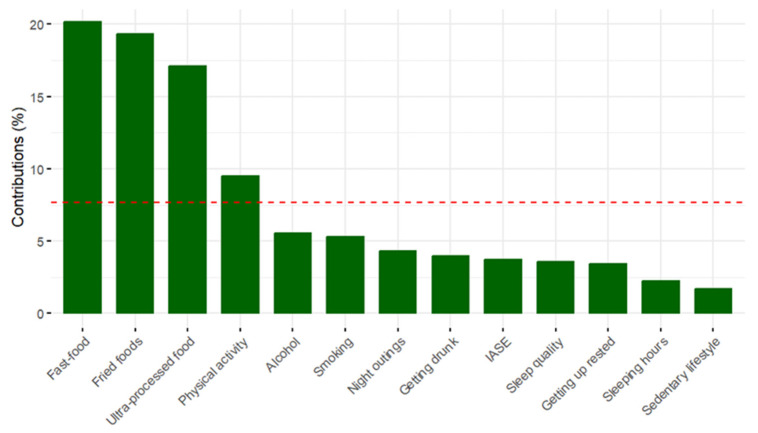
Graph of the contribution of the variables to dimension 1 of the model. (NOTE: The red discontinuous line represents the average contribution of all variables to the first dimension.).

**Figure 4:**
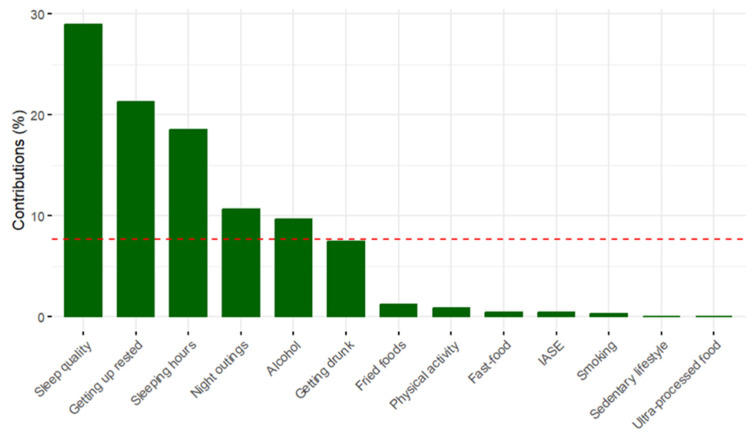
Graph of the contribution of the variables to dimension 2 of the model. (NOTE: The red discontinuous line represents the average contribution of all variables to the second dimension.).

Figure 3 shows the contribution of the variables to dimension 1 of the model. The main variables in the first dimension are: fast food, fried food, ultra-processed food and physical activity. Figure 4 illustrates the graph of the contribution of the variables to dimension 2 of the model. It can be seen how the variables Quality of sleep, Waking up rested, Sleeping hours, Night out and Alcohol are the variables that contribute most to the second dimension.

**Figure 5:**
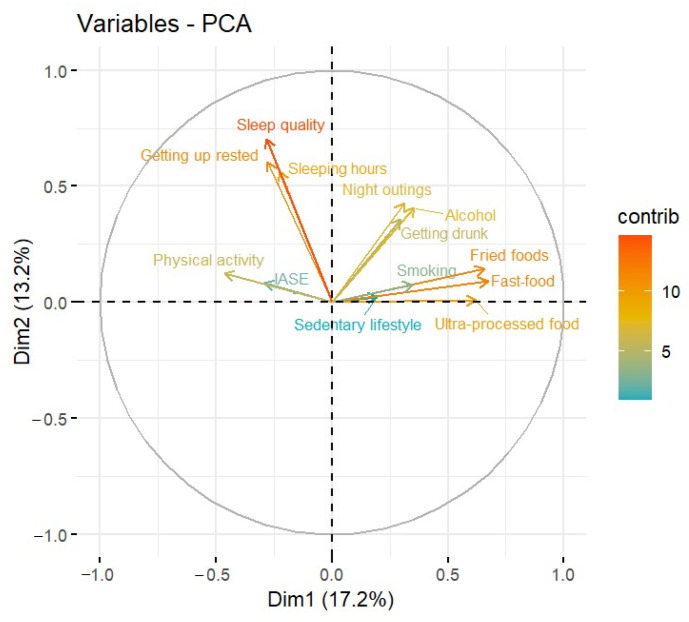
Graphical representation of the PCA models in 2 dimensions.

The contribution of the variables in dimension 1 can also be seen in Figure 5 by looking at the length of the arrows of these variables, their proximity to the X-axis and their color (a more of red or orange color indicates a higher contribution of a variable to the model, while a more of bluish or green color indicates a lower contribution to the model). The same applies to the contribution of dimension 2 that is reflected in Figure 5, considering the proximity of these rows to the Y-axis, rather than the X-axis, and and the length of the arrows, which are longer than the others.

The PCA graph shows the relationship between variables. As can be seen by their red and orange coloring, variables such as Quality of sleep, Waking up rested, Fried food and Fast food contribute the most to the model. On the other hand, as the color legend indicates, the variables colored in blue, Sedentary behavior and IASE, contribute the least. For the other variables studied, the contribution is moderate, with colors ranging from yellow to very light green. However, Hours of sleep and Ultra-processed food (shown in yellow) contribute slightly more to the model than variables Frequency of physical activity, Night out, Alcohol, Binge drinking and Smoking (shown in olive green). On the other hand, there is no relationship between the variables Quality of sleep, Waking up rested and Sleeping hours and the variables Fried food, Fast food and Ultra-processed food, as the arrows form approximately a 90° angle.

Consequently, what differs between the young adult Spanish population is the sleep quality, getting up rested and the consumption of fried foods and fast food.

Secondly, a linear regression was performed to study the linear relationship between the IASE (predicted) variable and the diet and habits (predicting) variables. Table 5 displays the coefficients of this linear regression. It is crucial to note that this model has an R-squared value of 0.05231, suggesting that the habits variables explain 5.23% of the variability of IASE. Therefore, the conclusions obtained have low forcefulness. The significant coefficient variables are Physical activity, fast food, Ultra-processed food, Sleep quality, Smoking, Alcohol and Getting drunk. In terms of interpretation, it can be observed that an increase in physical activity is associated with a tiny increase in IASE while the consumption of ultra-processed foods and smoking is negatively associated with IASE. Additionally, fast-food consumption, sleep quality and night outings also have a negative effect on IASE.

## 4. Discussion

In the present study, the influence of sociodemographic variables on some nutritional and lifestyle factors was considered. Firstly, focusing on gender, the results obtained indicate that men consume more coffee and eat more frequently fried and ultra-processed foods. This could imply that men have less healthy dietary habits than women. In fact, high consumption of fried foods increases the risk of obesity, heart disease [30] type 2 diabetes [31] and other health problems. Frequent consumption of ultra-processed foods has also been linked to various adverse health effects, such as obesity, metabolic disorders and an increased risk of chronic diseases [32,33]. However, when analyzing the IASE, no significant differences were found between sexes, nor were there differences in fast food consumption, Therefore, the difference between sexes in terms of feeding does not seem to be very pronounced, at least in the age range considered in this study. This result can be used as a guide when designing food education and awareness-raising policies, indicating the possibility of using a unified language for both sexes.

In terms of social habits, a significant distinction can be found in alcohol consumption. It was observed that men consumed alcohol more frequently than women and got drunk more often. Therefore, it may be important to consider awareness-raising actions of the negative effects of alcohol consumption, specifically targeting the male audience. In fact, previous studies have already observed that men consume alcohol more frequently than women and have higher rates of alcohol-related problems such as intoxication and alcohol use disorders [34]. In these actions, it will be important to focus on social and cultural factors which appear to be factors that partly explain these differences [34]. Peer pressure may also influence drinking patterns, and men may be more likely to consume alcohol to cope with stress due to social expectations that discourage them from openly expressing their vulnerability or seeking emotional support [35].

Another important difference is found in the time spent on physical activity, with males spending notably more time on physical activity than females. This result aligns with the general trend that men tend to devote more time to physical activity than women, on average [36,37]. Regular physical activity has numerous health benefits, including strengthening the heart, improving blood circulation, reducing the risk of cardiovascular diseases such as heart attacks and strokes [38] It can also help to control body weight by burning calories and maintaining a healthy metabolism [39]. Additionally, it has positive effects on mental well-being by reducing symptoms of anxiety and depression, improving mood and promoting overall mental health [40]. For all these reasons, another point on which a training and awareness-raising campaign could have an impact is the promotion of sports practices among women. Regular physical activity is quite important because it can also improve sleep quality, aiding in falling asleep more quickly and enjoying a more restful sleep [41]. This might explain the differences found on the variables sleep quality and feeling rested upon waking, with men reporting better sleep quality and a more refreshed awakening, despite no differences in the duration of sleep.

While dietary differences by sex did not seem to be determinant, they were notable concerning the age of the population. The older age group seems to have better nutrition, as evidenced by the higher IASE score. Also aligned in terms of health, the consumption of fast food, fried food and ultra-processed food, which are harmful to health, is less frequent among older individuals.

It is commonly observed that adults tend to be more conscious of their nutrition compared to younger people. This may be due to higher levels education and increased awareness of the importance of nutrition and its impact on overall health [42]. As people aged, they often become more aware of the relationship between their diet and various health issues, motivating them to prioritize healthier food choices. Furthermore, diet-related chronic diseases are more prevalent among the older age group [43], which may lead to greater awareness of health status and potential risks, encouraging them to adopt healthier eating habits and to follow specific dietary guidelines. Lastly, adults often have more access to resources such as nutritional information, culinary skills and financial means to afford healthier food choices.

Along these lines, promoting food education could be interesting, primarily in schools, but also at the university level. Integrating seminars or interdisciplinary subjects into curricula to provide young people with in-depth knowledge of nutrition, cooking techniques or healthy habits would undoubtedly help them in making better food choices and positively impact their nutritional health.

While nutrition in adults is generally healthier than in younger people, it seems to follow an opposite trend in terms of sleep habits. People over 30 years of age sleep, on average, for less time, wake up feeling less rested and rate the quality of their sleep as lower than younger people. With age, natural changes in sleep patterns may occur. Older adults may experience a change in their circadian rhythm, causing them to go to bed and wake up earlier [44]. Hormonal changes, such as decreased melatonin production, can affect sleep quality and make it difficult to fall asleep and maintain sleep [45,46]. These physiological changes can hardly be reversed, but other factors may contribute to poorer sleep patterns and can be addressed at both the public health and individual levels. Adults often also have multiple responsibilities including work, family obligations, caregiver duties and other factors that can contribute to sleep deprivation and perceived sleep difficulties. Seeking moments of rest and disconnection to reduce the level of stress, trying to reduce social interactions, spending time in natural settings, etc., are small actions that everyone can take to improve sleep patterns and reduce fatigue [5,47,48].

Regarding the educational level of the surveyed individuals, we found significant differences in all the variables except for water consumption and the frequency of nights out. Those with higher education have a better healthy nutrition index, a lower frequency of consumption of fast food, fried food and ultra-processed food and a higher frequency of fish consumption. The results obtained are in line with the literature, where different studies have found a connection between schooling and improved nutrition [49,50]. It confirms that a higher level of education increases people’s awareness of the importance of a balanced diet and the potential health risks associated with incorrect or poor nutrition. Moreover, individuals with higher education tend to be more competent in interpreting nutrition labels, understanding dietary guidelines and critically evaluating nutritional information [51]. This positively impact on their food choices and the adoption of better culinary and nutritional habits. In the light of these results, it may be useful at state, regional and local levels to think of training actions especially focused on those with lower levels of education to facilitate their access to reliable and accurate nutrition information. Furthermore, at the food industry and legislative levels, it seems to be interesting to rethink how the information on components and micronutrients is provided on product labels and packaging. Presenting this information in a clearer and simpler way could facilitate understanding for those without higher education.

A reflection should also be made on the quality of sleep and rest in relation to the level of education, generally related to the type of work carried out by individuals. People with higher education sleep more hours on average, report better sleep quality and claim to wake up more rested than people with basic education. Generally, individuals with basic studies are more likely to perform a greater proportion of low-skilled physical and manual labor, while those with higher education will tend to perform more specialized intellectual work. The type of work, whether intellectual or physical, may have different effects on fatigue levels and sleep quality [52,53]. Intellectual work provides mental stimulation and engages the mind in challenging tasks, which can promote alertness and cognitive activity [54]. In contrast, physical or manual labor may involve repetitive movements, heavy lifting or prolonged physical exertion, leading to physical tiredness and muscle fatigue [55]. Intellectual work does not usually exert the same level of physical strain, which reduces physical exhaustion. It is important that companies, in collaboration with occupational safety agencies, study all workplaces to ensure that all workers have healthy working conditions that they can maintain over time without compromising their health.

Analogous reflections to those raised for the level of education can be applied to the level of income. As the data obtained in this investigation suggest, previous studies have also shown how people with higher incomes tend to have better nutrition and access to healthier food [56,57]. One possible explanation for this disparity can be found in the affordability of healthier foods, in fact foods such as fresh fruits and vegetables, lean proteins and whole grains can sometimes be more expensive than processed or unhealthy alternatives [58]. People with higher incomes have more financial resources to afford these healthier foods and may face fewer constraints in purchasing nutritious ingredients. Generally, higher income urban areas tend to have better access to supermarkets, farmers’ markets and specialty stores that offer a wide variety of fresh and nutritious foods compared to low-income neighborhoods with limited access to healthy foods [59]. Higher incomes can also contribute to reduced worries and potentially improved sleep for several reasons. They often provide people with greater financial stability and security, allowing them to meet their basic needs, pay bills and cope with unexpected expenses. Having a sense of financial security can alleviate worry and reduce stress related to financial matters, which can positively impact on sleep quality [60]. On the other hand, individuals with higher incomes may have access to resources and services that can support their well-being, including mental health services, relaxation therapies and stress management programs [61]. Utilizing these resources can help to reduce worry and promote better sleep. From these observations, individuals with a lower educational and economic level seem to be more vulnerable subjects to which more attention should be paid in terms of public health, as they are generally more likely to adopt unhealthy nutrition and lifestyle habits.

In an overall view, for all the analyzed groups, we found the need for dietary changes. Classifying the IASE on a three-value scale, with criteria analogous to those used by Norte and Ortiz in their study [27], we assign the classification of ‘Healthy’ for an IASE score between 58.4 and 73, ‘Needs changes’ for an IASE between 36.5 and 58.4 and ‘Unhealthy’ for an IASE below 36.5. In the present study, we found that for all the sociodemographic variables explored, the mean nutritional index obtained falls within the range where changes are needed.

Regarding the practice of physical activity, it is known that the recommended amount can vary according to factors such as age, overall health and individual fitness goals. However, the World Health Organization (WHO) provides general guidelines for adults aged 18–64 years, suggesting at least 150 min of moderate-intensity aerobic activity throughout the week [62]. This can be achieved through activities such as brisk walking, cycling, swimming or dancing. Alternatively, at least 75 min of vigorous-intensity aerobic activity per week can be proposed. For all the groups studied, we can see that the weekly time dedicated to physical activity is very close to and, in several cases, exceeds these recommendations, allowing us to affirm that this habit is healthy in the studied population.

Finally, if we consider which foods and lifestyle habits have the most profound impact on overall health and therefore on what should be given more attention, we can find some clues in the results of the PCA analysis. In Figure 5, we can see that the number of hours spent resting and, above all, the quality of sleep has a high impact on health (indicated by red arrows), while in food, the most significant impact is associated by the consumption of fried, ultra-processed or fast-food items. On the other hand, the number of hours a person spends sitting and smoking seems to contribute less in terms of health.

### This Study’s Strengths and Limitations

The large sample size (n = 18,070) and geographic coverage (data collected from all regions of Spain and the islands) are undoubtedly the strengths of this study. With a large and geographically heterogeneous sample size, error is reduced, and high statistical power can be achieved. Simultaneously, potential geographical bias is reduced, increasing the external validity of the research and favoring the application of results to a wider population.

Additionally, the potential of the instrument obtained cannot be overlooked. The multifactorial nature of the NutSo-HH scale and its capacity to measure different aspects of healthy habits, make it a useful instrument to support the screening of the nutritional and social habits of a population.

However, the type of sampling used represents one of this study´s weaknesses. The dissemination of the questionnaire through networks revealed a certain self-selection bias, as many of the people who collaborated in the dissemination, as well as some of those who answered the survey, were interested in nutrition and health, possessing some training and knowledge in this field. To limit this bias, an important effort was made to distribute the survey also through other channels, including collaboration with establishments or associations carefully selected to cover the whole Spanish territory and to gather a heterogeneous public.

Another bias that should be highlighted is the gender bias, with 82.29% of respondents being female. It is not uncommon to observe a higher participation rate among women in studies related to nutrition and health. Aware of this trend, a specific effort was made to recruit male participants for this study, eventually achieving a broadly representative sample of 3200 men.

Self-report bias is a major challenge in cohort studies using nutrition surveys. Self-report bias can occur when participants provide inaccurate or biased information about their food intake and dietary patterns. Although this limitation cannot be overcome, the results obtained in this study largely align with those highlighted by previous studies, providing some confidence in the conclusions that can be reached.

## 5. Conclusions

In general, the Spanish young adult population demonstrates healthy habits, although there is room for improvement in certain aspects related to nutrition. Their weekly physical activity aligns with the health recommendations set by the World Health Organization.

Gender differences are not prominent in most nutritional habits. However, men tend to engage in more sports activities and have better sleep quality, while consuming more alcohol.

When comparing adults to young people, adults exhibit better nutritional habits. However, their quality of rest is poorer, they engage in less physical activity and tend to have a more sedentary lifestyle.

Overall, the education and purchasing power of the Spanish population significantly influence their lifestyle habits. Education fosters knowledge and awareness regarding the importance of leading a healthy lifestyle, while purchasing power provides the necessary resources to make such choices. These two factors work hand in hand to shape dietary preferences, level of physical activity and overall well-being.

## Figures and Tables

**Table 1:** Categorization of variables not included in the IASE.

Variable	Category	Score
Sleeping hours	<6 h for night	1
	Between 6 and 7 h	2
	Between 7 and 8 h	3
	More than 8 h	4
Sleep quality	0 and 1	1
	2	2
	3	3
	4 and 5	4
Getting up rested	Never	1
	Very seldom and sometimes	2
	Frequently and almost always	3
	Always	4
Consumption of fast food, fried and ultra-processed dishes	Never	1
	Very seldom (2 times a month maximum)	2
	Once a week	3
	Several times a week	4
Smoking	Nonsmoker	1
	Light smoker (less than 5 cigarettes per day)	2
	Moderate smoker (6–15 cigarettes per day)	3
	Severe smoker (more than 16 cigarettes per day)	4
Night outings	Never and sporadically	1
	Between 1 and 2 nights a week	2
	More than 3 times a week	3
	Every day	4
Getting Drunk	Never or less than once a month	1
	Monthly	2
	Weekly	3
	Daily or almost daily	4
Alcohol consumption	Never and once a month	1
	2 to 4 times a month	2
	2 to 3 times a week	3
	4 to 5 times a week and every day	4
Sedentary lifestyle	Less than 7 h	1
	Between 7 and 9 h	2
	Between 9 and 11 h	3
	More than 11 h	4

**Table 2:** Sociodemographic characteristics of the sample.

	Total Sample	Male	Female	Fisher’s Test
	(n = 18,070)	n = 3200 (17.71%)	n = 14,870 (82.29%)	
Age (years)				
18–30	9808 (57.42%)	1854 (57.9%)	7954 (53.5%)	0.000 **
31–45	8262 (42.58%)	1346 (42.1%)	6919 (46.5%)	
Level of education				
Basic education	5782 (33.85%)	1236 (38.6%)	4546 (30.6%)	0.000 **
Higher education	12,288 (66.15%)	1964 (61.4%)	10,324 (69.4%)	
Income level				
Low	8448 (49.46%)	1430 (44.7%)	7018 (47.2%)	0.001 **
Medium–high	8032 (50.54%)	1527 (47.7%)	6505 (43.7%)	
Body Mass Index (BMI)	23.73 ± 44.67	24.74 ± 44.15	23.51 ± 44.75	

Age, level of education, Income level. ** *p* < 0.01.

**Table 3:** Mean and standard deviation of nutrition and habits with respect to sex, age, educational level and income level.

	Sex (Mean ± SD)		Age (Mean ± SD)		Educational Level (Mean ± SD)		Income Level (Media ± SD)	
Habits Variables	Female	Male	Young (18–30 Years)	Adults (31–45 Years)	Basic Education	High Education	Low Incomes	Medium–High Incomes
Healthy Eating Index (IASE)	53.70 ± 9.81	53.53 ± 10.01	53.32 ± 9.71	54.09 ± 9.98	52.00 ± 10.38	54.46 ± 9.48	52.75 ± 10.13	54.70 ± 9.49
Sedentary lifestyle	1.60 ± 0.71	1.61 ± 0.88	1.65 ± 0.87	1.55 ± 0.82	1.55 ± 0.83	1.63 ± 0.86	1.54 ± 0.83	1.66 ± 0.85
Physical activity (min)	138.2 ± 159.9	266.3 ± 223.3	174.1 ± 188.9	145.2 ± 166.5	165.5 ± 195.7	158.7 ± 171.5	161.9 ± 182.7	158.7 ± 175.5
Fast-food consumption	2.45 ± 0.57	2.48 ± 0.59	2.53 ± 0.74	2.37 ± 0.76	2.51 ± 0.76	2.43 ± 0.75	2.48 ± 0.76	2.43 ± 0.75
Consumption of fried foods	2.25 ± 0.81	2.31 ± 0.86	2.35 ± 0.84	2.14 ± 0.79	2.37 ± 0.87	2.21 ± 0.79	2.26 ± 0.84	2.24± 0.80
Consumption of ultra-processed food	2.42 ± 0.94	2.31 ± 0.95	2.45 ± 0.93	2.34 ± 0.95	2.48 ± 0.96	2.36 ± 0.93	2.44 ± 0.95	2.36 ± 0.93
Sleeping hours	2.56 ± 0.73	2.56 ± 0.51	2.66 ± 0.69	2.45 ± 0.74	2.53 ± 0.75	2.55 ± 0.71	2.55 ± 0.74	2.57 ± 0.71
Getting up rested	2.51 ± 0.58	2.58 ± 0.58	2.54 ± 0.57	2.51 ± 0.58	2.48 ± 0.59	2.55 ± 0.57	2.50 ± 0.59	2.56 ± 0.57
Sleep quality	3.39 ± 1.02	3.51 ± 0.96	3.50 ± 0.98	3.30 ± 1.04	3.34 ± 1.03	3.44 ± 1.00	3.34 ± 1.02	3.47 ± 0.99
Smoking	1.22 ± 0.60	1.21 ± 0.61	1.21 ± 0.59	1.22 ± 0.62	1.30 ± 0.70	1.19 ± 0.54	1.25 ± 0.64	1.18 ± 0.56
Alcohol consumption	1.67 ± 0.80	1.82 ± 0.88	1.69 ± 0.77	1.71 ± 0.86	1.61 ± 0.79	1.74 ± 0.82	1.64 ± 0.79	1.76 ± 0.84
Getting drunk	1.05 ± 0.27	1.12 ± 0.41	1.08 ± 0.32	1.05 ± 0.27	1.07 ± 0.32	1.06 ± 0.29	1.06 ± 0.30	1.06 ± 0.29
Night outings	1.20 ± 0.44	1.24 ± 0.48	1.30 ± 0.51	1.09 ± 0.31	1.21 ± 0.46	1.20 ± 0.44	1.20 ± 0.44	1.19 ± 0.44

**Table 4:** Analysis of the differences in the variables of nutrition and habits with respect to sex, age, educational level and income level.

	Sex				Age			Educational Level			Income Level	
	ES	95% CI for Difference of Means		ES	95% CI for Difference of Means		ES	95% CI for Difference of Means		ES	95% CI for Difference of Means	
	Cohen’s			Cohen’s			Cohen´s			Cohen´s		
Habits		Lower	Upper		Lower	Upper		Lower	Upper		Lower	Upper
Variables												
Healthy Eating Index (IASE)	0.02	−0.2	0.55	−0.08	−1.05	−0.48	−0.25	−2.76	−2.15	−0.2	−2.24	−1.64
Sedentary lifestyle	−0.01	−0.04	0.02	0.12	0.08	0.13	−0.1	−0.11	−0.06	−0.14	−0.14	−0.09
Physical activity	−0.74	−134.7	−121.5	0.16	23.71	34.19	0.04	1.23	12.46	0.02	−2.31	8.65
Fast-food consumption	−0.04	−0.06	0	0.22	0.15	0.19	0.11	0.06	0.11	0.07	0.03	0.08
Consumption of fried foods	−0.07	−0.09	−0.03	0.26	0.19	0.23	0.19	0.13	0.18	0.02	−0.01	0.04
Consumption of ultra-processed food	0.12	0.08	0.15	0.13	0.09	0.15	0.12	0.09	0.15	0.09	0.05	0.11
Sleeping hours	0	−0.03	0.03	0.29	0.19	0.23	−0.06	−0.07	−0.02	−0.03	−0.04	0
Getting up rested	−0.11	−0.08	−0.04	0.06	0.02	0.05	−0.12	−0.09	−0.05	−0.1	−0.07	−0.04
Sleep quality	−0.12	−0.16	−0.08	0.2	0.18	0.23	−0.1	−0.14	−0.07	−0.12	−0.15	−0.09
Smoking	0.02	−0.01	0.04	−0.01	−0.02	0.01	0.2	0.1	0.14	0.11	0.05	0.09
Alcohol consumption	−0.19	−0.18	−0.12	−0.02	−0.04	0.01	−0.16	−0.15	−0.1	−0.14	−0.14	−0.09
Getting drunk	−0.22	−0.08	−0.06	0.1	0.02	0.04	0.04	0	0.02	0	−0.01	0.01
Night outings	−0.1	−0.06	−0.03	0.48	0.19	0.22	0.03	0	0.03	0.02	0	0.02

Note Student’s *t*-test.

**Table 5:** Coefficients of the linear regression and their significance.

Habits Variables	Coefficients	*p*-Value
Intercept	57.646	<2 × 10^−16^
Sedentary lifestyle	−0.056	0.514
Physical activity (min)	0.004	<2 × 10^−16^
Fast-food consumption	−0.700	2.35 × 10^−106^
Consumption of fried foods	−0.069	0.493
Consumption of ultra-processed food	−0.495	9.91 × 10^−9^
Sleeping hours	0.009	0.934
Getting up rested	0.112	0.411
Sleep quality	0.532	8.82 × 10^−11^
Smoking	−2.584	<2 × 10^−16^
Alcohol consumption	0.750	4.11 × 10^−15^
Getting drunk	−1.060	3.72 × 10^−5^
Night outings	−0.445	0.010

## Data Availability

The data presented in this study are available upon request to the corresponding author.

## References

[1] Svalastog A.L., Donev D., Kristoffersen N.J., Gajović S. (2017). Concepts and Definitions of Health and Health-Related Values in the Knowledge Landscapes of the Digital Society. Croat. Med. J..

[2] Oleribe O.O., Ukwedeh O., Burstow N.J., Gomaa A.I., Sonderup M.W., Cook N., Waked I., Spearman W., Taylor-Robinson S.D. (2018). Health: Redefined. Pan Afr. Med. J..

[3] Burgner D., Jamieson S.E., Blackwell J.M. (2006). Genetic Susceptibility to Infectious Diseases: Big Is Beautiful, but Will Bigger Be Even Better?. Lancet Infect. Dis..

[4] Saarloos D., Kim J.E., Timmermans H. (2009). The Built Environment and Health: Introducing Individual Space-Time Behavior. Int. J. Environ. Res. Public Health.

[5] Jimenez M.P., Deville N.V., Elliott E.G., Schiff J.E., Wilt G.E., Hart J.E., James P. (2021). Associations between Nature Exposure and Health: A Review of the Evidence. Int. J. Environ. Res. Public Health.

[6] Williams J. (2022). Where We Work, Play, And Live: Health Equity and the Physical Environment. North Carol. Med. J..

[7] O’Connor D.B., Thayer J.F., Vedhara K. (2021). Stress and Health: A Review of Psychobiological Processes. Annu. Rev. Psychol..

[8] Horenstein A., Heimberg R.G. (2020). Anxiety Disorders and Healthcare Utilization: A Systematic Review. Clin. Psychol. Rev..

[9] Gaynes B.N., Burns B.J., Tweed D.L., Erickson P. (2002). Depression and Health-Related Quality of Life. J. Nerv. Ment. Dis..

[10] Smith K.J., Victor C. (2022). The Association of Loneliness With Health and Social Care Utilization in Older Adults in the General Population: A Systematic Review. Gerontologist.

[11] Farhud D.D. (2015). Impact of Lifestyle on Health. Iran. J. Public Health.

[12] Marcos-Delgado A., Hernández-Segura N., Fernández-Villa T., Molina A.J., Martín V. (2021). The Effect of Lifestyle Intervention on Health-Related Quality of Life in Adults with Metabolic Syndrome: A Meta-Analysis. Int. J. Environ. Res. Public Health.

[13] Santos L. (2022). The Impact of Nutrition and Lifestyle Modification on Health. Eur. J. Intern. Med..

[14] Zeinali F., Habibi N., Samadi M., Azam K., Djafarian K. (2016). Relation between Lifestyle and Socio-Demographic Factors and Body Composition among the Elderly. Glob. J. Health Sci..

[15] Vajdi M., Nikniaz L., Asl A.M.P., Farhangi M.A. (2020). Lifestyle Patterns and Their Nutritional, Socio-Demographic and Psychological Determinants in a Community-Based Study: A Mixed Approach of Latent Class and Factor Analyses. PLoS ONE.

[16] Lee H., Lee D., Guo G., Harris K.M. (2011). Trends in Body Mass Index in Adolescence and Young Adulthood in the United States: 19592002. J. Adolesc. Health.

[17] Nelson M.C., Story M., Larson N.I., Neumark-Sztainer D., Lytle L.A. (2008). Emerging Adulthood and College-Aged Youth: An Overlooked Age for Weight-Related Behavior Change. Obesity.

[18] Desbouys L., De Ridder K., Rouche M., Castetbon K. (2019). Food Consumption in Adolescents and Young Adults: Age-Specific Socio-Economic and Cultural Disparities (Belgian Food Consumption Survey 2014). Nutrients.

[19] Nagai M. (2020). Relationships among Lifestyle Awareness, Age, and Lifestyle-Related Diseases in Healthy Japanese Community Residents. Asian Pac. Isl. Nurs. J..

[20] Mollborn S., Lawrence E.M., Hummer R.A. (2020). A Gender Framework for Understanding Health Lifestyles. Soc. Sci. Med..

[21] Eguchi E., Iso H., Honjo K., Yatsuya H., Tamakoshi A. (2017). No Modifying Effect of Education Level on the Association between Lifestyle Behaviors and Cardiovascular Mortality: The Japan Collaborative Cohort Study. Sci. Rep..

[22] Moagi M., Mulaudzi M., Van Der Wath A. (2019). Support Programs for Students at Higher Education Institutions in South Africa: An Appreciative Inquiry Study on Managing Alcohol Abuse. J. Subst. Use.

[23] Ahmadi A., Roosta F. (2015). Health Knowledge and Health Promoting Lifestyle Among Women of Childbearing Age in Shiraz. Women’s Health Bull..

[24] Brancato G., Macchia S., Murgia M., Signore M., Simeoni G., Blanke K., Hoffmeyer-Zlotnik J. (2006). Handbook of Recommended Practices for Questionnaire Development and Testing in the European Statistical System.

[25] Ventura-León J.L., Caycho-Rodríguez T. (2017). El Coeficiente Omega: Un Método Alternativo Para La Estimación de La Confiabilidad. Rev. Latinoam. Cienc. Soc. Niñez Juv..

[26] Renta Anual Neta Media Por Tipo de Hogar. Renta Relativa de Las Personas Mayores. https://www.ine.es/ss/Satellite?param1=PYSDetalle&c=INESeccion_C&param3=1259924822888&p=%5C&pagename=ProductosYServicios%2FPYSLayout&cid=1259925949467&L=1.

[27] Norte Navarro A.I., Ortiz Moncada R. (2011). Calidad de La Dieta Española Según El Índice de Alimentación Saludable. Nutr. Hosp..

[28] Salvador Castell G., Ribas Barba L., Pérez Rodrigo Ayuntamiento de Bilbao C., Arija Val Universitat Rovira Virgili V., Senc R., García Perea A., Román Martínez Álvarez J., Mᵃ Ortega Anta R., Tur Marí J.A., Aranceta Bartrina J. (2004). Guía de La Alimentación Saludable.

[29] Hopkins W.G., Marshall S.W., Batterham A.M., Hanin J. (2009). Progressive Statistics for Studies in Sports Medicine and Exercise Science. Med. Sci. Sports Exerc..

[30] Gadiraju T.V., Patel Y., Gaziano J.M., Djoussé L. (2015). Fried Food Consumption and Cardiovascular Health: A Review of Current Evidence. Nutrients.

[31] Cahill L.E., Pan A., Chiuve S.E., Sun Q., Willett W.C., Hu F.B., Rimm E.B. (2014). Fried-Food Consumption and Risk of Type 2 Diabetes and Coronary Artery Disease: A Prospective Study in 2 Cohorts of US Women and Men. Am. J. Clin. Nutr..

[32] Nardocci M., Polsky J.Y., Moubarac J.C. (2021). Consumption of Ultra-Processed Foods Is Associated with Obesity, Diabetes and Hypertension in Canadian Adults. Can. J. Public Health.

[33] Pagliai G., Dinu M., Madarena M.P., Bonaccio M., Iacoviello L., Sofi F. (2021). Consumption of Ultra-Processed Foods and Health Status: A Systematic Review and Meta-Analysis. Br. J. Nutr..

[34] White A.M. (2020). Gender Differences in the Epidemiology of Alcohol Use and Related Harms in the United States. Alcohol. Res..

[35] Sudhinaraset M., Wigglesworth C., Takeuchi D.T. (2016). Social and Cultural Contexts of Alcohol Use: Influences in a Social–Ecological Framework. Alcohol. Res..

[36] Craft B.B., Professor of Psychology A., Carroll H.A., Faculty A., Kathleen Lustyk M.B. (2014). Gender Differences in Exercise Habits and Quality of Life Reports: Assessing the Moderating Effects of Reasons for Exercise. Int. J. Lib. Arts Soc. Sci..

[37] Azevedo M.R., Araújo C.L.P., Reichert F.F., Siqueira F.V., da Silva M.C., Hallal P.C. (2007). Gender Differences in Leisure-Time Physical Activity. Int. J. Public Health.

[38] Nystoriak M.A., Bhatnagar A. (2018). Cardiovascular Effects and Benefits of Exercise. Front. Cardiovasc. Med..

[39] Cox C.E. (2017). Role of Physical Activity for Weight Loss and Weight Maintenance. Diabetes Spectr..

[40] Sharma A., Madaan V., Petty F.D. (2006). Exercise for Mental Health. Prim. Care Companion CNS Disord..

[41] Kline C.E. (2014). The Bidirectional Relationship between Exercise and Sleep: Implications for Exercise Adherence and Sleep Improvement. Am. J. Lifestyle Med..

[42] Sun Y., Dong D., Ding Y. (2021). The Impact of Dietary Knowledge on Health: Evidence from the China Health and Nutrition Survey. Int. J. Environ. Res. Public Health.

[43] Jaul E., Barron J. (2017). Age-Related Diseases and Clinical and Public Health Implications for the 85 Years Old and Over Population. Front. Public Health.

[44] Gadie A., Shafto M., Leng Y., Kievit R.A., Cam-CAN (2017). How Are Age-Related Differences in Sleep Quality Associated with Health Outcomes? An Epidemiological Investigation in a UK Cohort of 2406 Adults. BMJ Open.

[45] Foster R.G. (2020). Sleep, Circadian Rhythms and Health. Interface Focus..

[46] Zisapel N. (2018). New Perspectives on the Role of Melatonin in Human Sleep, Circadian Rhythms and Their Regulation. Br. J. Pharmacol..

[47] daSilva A.W., Huckins J.F., Wang W., Wang R., Campbell A.T., Meyer M.L. (2021). Daily Perceived Stress Predicts Less Next Day Social Interaction: Evidence From a Naturalistic Mobile Sensing Study. Emotion.

[48] Kondo M.C., Jacoby S.F., South E.C. (2018). Does Spending Time Outdoors Reduce Stress? A Review of Real-Time Stress Response to Outdoor Environments. Health Place.

[49] Rippin H.L., Hutchinson J., Greenwood D.C., Jewell J., Breda J.J., Martin A., Rippin D.M., Schindler K., Rust P., Fagt S. (2020). Inequalities in Education and National Income Are Associated with Poorer Diet: Pooled Analysis of Individual Participant Data across 12 European Countries. PLoS ONE.

[50] Azizi Fard N., De Francisci Morales G., Mejova Y., Schifanella R. (2021). On the Interplay between Educational Attainment and Nutrition: A Spatially-Aware Perspective. EPJ Data Sci..

[51] Miller L.M.S., Cassady D.L. (2015). The Effects of Nutrition Knowledge on Food Label Use. A Review of the Literature. Appetite.

[52] Åkerstedt T., Wright K.P. (2009). Sleep Loss and Fatigue in Shift Work and Shift Work Disorder. Sleep. Med. Clin..

[53] Martins A.J., Vasconcelos S.P., Skene D.J., Lowden A., De Castro Moreno C.R. (2016). Effects of Physical Activity at Work and Life-Style on Sleep in Workers from an Amazonian Extractivist Reserve. Sleep. Sci..

[54] Mather M. (2020). How Do Cognitively Stimulating Activities Affect Cognition and the Brain Throughout Life?. Psychol. Sci. Public Interest.

[55] Bláfoss R., Sundstrup E., Jakobsen M.D., Brandt M., Bay H., Andersen L.L. (2019). Physical Workload and Bodily Fatigue after Work: Cross-Sectional Study among 5000 Workers. Eur. J. Public Health.

[56] French S.A., Tangney C.C., Crane M.M., Wang Y., Appelhans B.M. (2019). Nutrition Quality of Food Purchases Varies by Household Income: The SHoPPER Study. BMC Public Health.

[57] Ohri-Vachaspati P., Deweese R.S., Acciai F., Delia D., Tulloch D., Tong D., Lorts C., Yedidia M. (2019). Healthy Food Access in Low-Income High-Minority Communities: A Longitudinal Assessment-2009-2017. Int. J. Environ. Res. Public Health.

[58] Rao M., Afshin A., Singh G., Mozaffarian D. (2013). Do Healthier Foods and Diet Patterns Cost More than Less Healthy Options? A Systematic Review and Meta-Analysis. BMJ Open.

[59] Hilmers A., Hilmers D.C., Dave J. (2012). Neighborhood Disparities in Access to Healthy Foods and Their Effects on Environmental Justice. Am. J. Public Health.

[60] Ryu S., Fan L. (2023). The Relationship Between Financial Worries and Psychological Distress Among U.S. Adults. J. Fam. Econ. Issues.

[61] Carter H., Araya R., Anjur K., Deng D., Naslund J.A. (2021). The Emergence of Digital Mental Health in Low-Income and Middle-Income Countries: A Review of Recent Advances and Implications for the Treatment and Prevention of Mental Disorders. J. Psychiatr. Res..

[62] World Health Organization (2010). Global Recommendations on Physical Activity for Health.

